# A Single Locus Is Responsible for Salinity Tolerance in a Chinese Landrace Barley (*Hordeum vulgare* L.)

**DOI:** 10.1371/journal.pone.0043079

**Published:** 2012-08-20

**Authors:** Rugen Xu, Junmei Wang, Chengdao Li, Peter Johnson, Chao Lu, Meixue Zhou

**Affiliations:** 1 Jiangsu Provincial Key Laboratory of Crop Genetics and Physiology and Barley Research Institution of Yangzhou University, Yangzhou, China; 2 Institute of Crop and Nuclear Technology Utilization, Zhejiang Academy of Agricultural Sciences, Hangzhou, China; 3 Department of Agriculture and Food, Government of Western Australia, South Perth, Western Australia, Australia; 4 Tasmanian Institute of Agriculture, University of Tasmania, Kings Meadows, Tasmania, Australia; University of Umeå, Sweden

## Abstract

**Introduction:**

Salinity and waterlogging are two major abiotic stresses severely limiting barley production. The lack of a reliable screening method makes it very hard to improve the tolerance through breeding programs.

**Methods:**

This work used 188 DH lines from a cross between a Chinese landrace variety, TX9425 (waterlogging and salinity tolerant), and a Japanese malting barley, Naso Nijo (waterlogging and salinity sensitive), to identify QTLs associated with the tolerance.

**Results:**

Four QTLs were found for waterlogging tolerance. The salinity tolerance was evaluated with both a hydroponic system and in potting mixture. In the trial with potting mixture, only one major QTL was identified to associate with salinity tolerance. This QTL explained nearly 50% of the phenotypic variation, which makes it possible for further fine mapping and cloning of the gene. This QTL was also identified in the hydroponic experiment for different salt-related traits. The position of this QTL was located at a similar position to one of the major QTLs for waterlogging tolerance, indicating the possibility of similar mechanisms controlling both waterlogging and salinity tolerance.

**Conclusion:**

The markers associated with the QTL provided a unique opportunity in breeding programs for selection of salinity and waterlogging tolerance.

## Introduction

Salinity and waterlogging are major abiotic stresses affecting crop production in high rainfall areas as well as irrigated agricultural lands. High intensity irrigation in more than 260 million hectares of land leads often to waterlogging and salinisation which is reducing the existing area under irrigation by 1–2 per cent per annum [1]. Global climate change is also expected to increase the frequency and severity of flooding events in many regions world-wide [2]. As the world population rises to a projected 9.2 billion in 2050 from 6.9 billion in 2010, food production has to be increased by more than70% [3]. To meet the demand, developing waterlogging and salt-tolerant crops becomes an important management option for maintaining production on waterlogging and/or salt-affected soils.

Barley is one of the major cereal crops [4] and relatively tolerant to salt [5] but susceptible to waterlogging [6]. Even though significant variation exists among different genotypes for both salinity [7], [8] and waterlogging tolerance [9], [10], a very slow progress has been made on improving the tolerance to both stresses, due to the lack of reliable selection methods. Various screening methods have scored a variety of phenotypic parameters including relative water content, germination rate, coleoptile length, stem and radicle length, as well as dry and wet weight of roots and shoots [11], [12], [13], [14], [15], [16] for salinity tolerance, leaf chlorosis, plant survival, yield components and physiological traits [10], [17], [18], [19], [20], [21] for waterlogging tolerance. However, most of the above indices are not readily applicable in breeding programs and so efforts have recently focused on generating molecular markers linked to these traits.

There have been many reports on QTLs associated with salt tolerance related traits which include yield and agronomic traits [16], [22], the tolerance at the germination and seedling stages [12], plant survival [23], shoot sodium content [24] and salt exclusion [25]. Relatively less report on QTLs controlling waterlogging tolerance were reported. Li et al. [19] used leaf chlorosis, plant biomass reduction and plant survival as tolerance indices and identified several QTLs for waterlogging tolerance in two different double haploid populations (crosses between tolerant and susceptible varieties). QTLs were also reported for agronomic traits and yield components under waterlogging conditions [17].

Accurate phenotyping is crucial for accurately locating QTL associated with quantitative traits which are easily affected by environment, such as waterlogging [26]. To achieve this, we have developed reliable screening facilities for both waterlogging and salinity tolerance evaluation and have successfully identified QTLs for both salinity and waterlogging tolerance from different DH populations [23], [26]. In the current study, we used 551 DArT markers and 75 SSR markers to construct a high density genetic map and screened 188 DH lines to identify QTLs associated with waterlogging tolerance and salinity tolerance at the late seedling stage. A hydroponic system was also used to evaluate salinity tolerance to compare the effectiveness of different screening methods on identifying QTL for salinity tolerance.

## Materials and Methods

### Populations Used for QTL Analysis

A total of 188 F_1_-derived doubled-haploid (DH) lines were generated from a cross between TX9425 and Naso Nijo. TX9425 is a Chinese landrace barley variety and was originally introduced as a source of waterlogging tolerance [9], [10], and it also showed some unique agronomic traits [27] and disease resistance [28], [29]. Naso Nijo is a Japanese malting barley and was susceptible to waterlogging [9].

### Map Construction

Genomic DNA was extracted from the leaf tissue of three-week-old seedlings, based on a modified CTAB method described by Stein et al. (2001). 326 SSR markers were selected to genotype the population (http://www.genetics.org/cgi/content/full/156/4/1997/DC1). The genotyping was conducted following the procedure of Ramsey et al. (2000). Genomic representations and preparation of the “discovery arrays” and “polymorphism-enriched arrays” for DArT analysis were as explained by Wenzl et al [30]. After removing non-polymorphic and low quality markers, 551 DArT markers and 75 SSR markers were used for map construction. The number of markers on different chromosomes ranged from 31 on 6H to 195 on 7H.The software package JoinMap 4.0 [31] was used to construct a complete linkage map. The number of markers on different chromosomes ranged from 31 on 6H to 195 on 7H. The map was finally compared with two DArT consensus maps [32], [33] and 7 markers in the new map which were located on different linkage groups in the consensus maps had an “a” added after the bPb numbers.

**Figure 1:**
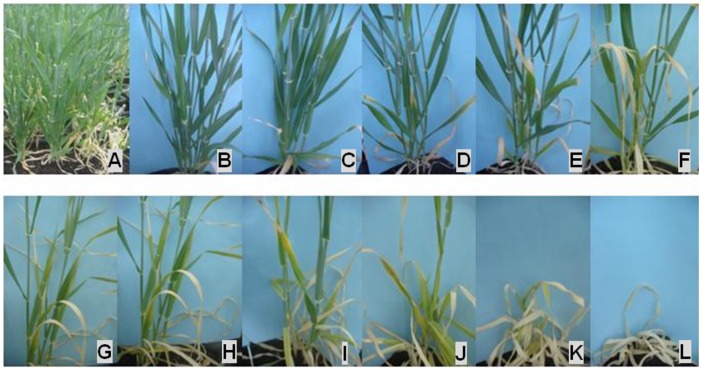
Range of phenotypes induced by salt stress at the booting stage in potting mixture experiment. A: part of the whole trial showing tolerant (left and middle) and susceptible lines (right) in the same trial; B to L: lines with scores of 0 (very tolerant) to 10 (very sensitive – all dead), respectively.

### Evaluating Salinity Tolerance with Potting Mixture

Seed of the parental varieties and the DH lines were sown in 40 L containers filled with a pine bark/loam based potting mix with premixed slow release fertiliser. The containers were located in a glasshouse at the Mt Pleasant Laboratories in Launceston, Tasmania. Each genotype comprised of three replicates, each of five seeds. The glasshouse experiment was performed during summer (sown on 11^th^ of February) in 2011 under natural light. The experiment was arranged as a randomized complete block design. The control experiment was not conducted since it has been proved that different varieties or DH lines grown in the same potting mixture but with no salt added showed no apparent symptoms of leaf chlorosis or wilting [23]. The salt treatment was similar to previously described [23]. Salt tolerance was assessed by combining scores for leaf chlorosis and plant survival when most of the DH lines reached booting stage (0 = no damage and 10 = all dead; scores between 0–5 are basically the level of leaf chlorosis and the number of dead leaves and score 6–10 are the percentage of plant survival as well as dead leaves and leaf chlorosis of survived plants). Figure 1 shows the differences among DH lines with low scores for lines showing less chlorosis or good survival and high scores for lines showing severe chlorosis and low survival.

**Figure 2:**
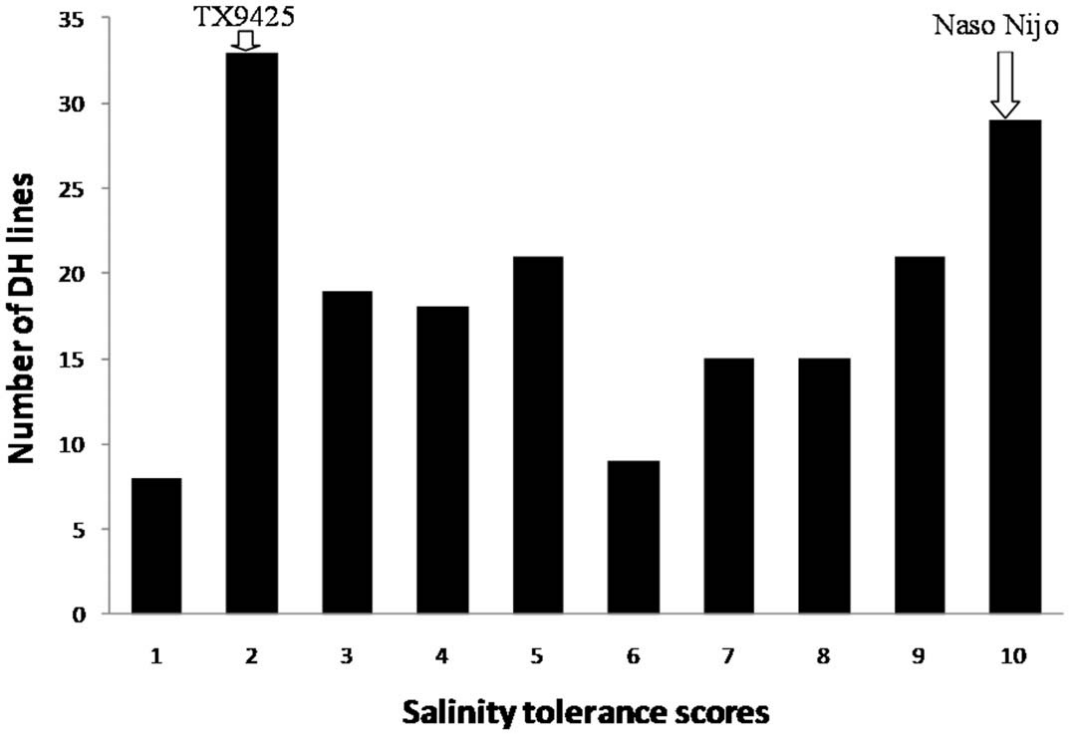
Frequency distribution for salinity tolerance at the booting stage of the DH lines derived from the cross of TX9425/Naso Nijo.

**Table 1:** ANOVA of salinity tolerance scores in a glasshouse potting mixture experiment and waterlogging tolerance in soil filled tank experiment.

Source of variance	df	SS	MS	F
Salinity				
DH lines	187	5156.6	27.6	5.7
Replication	2	120.1	60.1	12.3
Error	373	1814.7	4.9	
Waterlogging				
DH lines	187	2028.9	10.8	7.7
Replication	2	158.7	79.3	56.2
Error	374	527.6	1.4	

### Evaluating Salinity Tolerance with Hydroponic System

Holes with a diameter of 2 cm were made in foam plates (60×180 cm). The distance between the centre of holes was 10 cm. Nylon nets were placed on the bottom of the plates. The plates were then placed in two different pools filled with tap water. Two seeds of each line/parent were sown in each hole with three holes for each replication. One week after sowing, the water was replaced with nutrient solution which contained (mg/L): (NH_4_)_2_SO_4_: 48.2; MgSO_4_: 65.9; K_2_SO_4_: 15.9; KNO_3_: 18.5; Ca(NO_3_)_2_: 59.9; KH_2_PO_4_: 24.8; FeC_6_H_5_O_7_: 5; MnCl_2_⋅4H_2_O: 0.9; H_14_O_11_SZn: 0.11; CuSO_4_: 0.04; HBO_3_: 2.9; and H_2_MoO_4_: 0.01. The pH of the solution was 6.4. The nutrient solution or water was circulated through a pump to avoid hypoxia which may cause waterlogging damage to the plants. At the three leaf stage, salt (300 mM NaCl) was added to one of the pools. Three days after the treatment, susceptible lines started to show leaf chlorosis and wilting. Three weeks after salt treatment, salt treated seedlings were harvested and the following traits were recorded: the number of leaves per plant (LNs), the number of yellow leaves per plant (YLNs), root length (RLs), plant height (from bottom to top leaf, PHs), fresh weight of yellow leaves per plant (YLFWs), fresh weight of green leaves per plant (GLFWs), dry weight of yellow leaves per plant (YLDWs), dry weight of green leaves per plant (GLDWs), fresh weight of roots per plant (RFWs) and dry weight of roots per plant (RDWs). At the same time, the controls were also harvested and the number of leaves per plant (LNc), plant height (PHc), fresh weight of shoots (SFWc) and dry weight of shoots (SDWs) were recorded.

### Evaluation of Waterlogging Tolerance of the DH Lines in Soil

Each DH and parental line were sown in stainless steel tanks (200 cm×100 cm×85 cm) filled with soil from Cressy Research Station, Tasmania, Australia, where waterlogging occurs regularly, in 2010 at Mt Pleasant Laboratories in Launceston, Tasmania. Each line or parent variety contained 15 to 20 plants. Two replications were used. Starting from the three-leaf stage, all the lines were subjected to waterlogging (keeping the water level just above the soil surface) for nine weeks until susceptible lines died. A combined score system (plant waterlogging sensitivity, which is a combined score of leaf chlorosis and plant survival after waterlogging; 0 = no damage and 10 = all dead; scores between 0–5 are basically the level of leaf chlorosis and score 6–10 are the percentage of plant survival as well as leaf chlorosis of survived plants) [26] was used. The trial was sown in late April with waterlogging treatment staring from late May to August, which is similar to agricultural practices.

**Figure 3:**
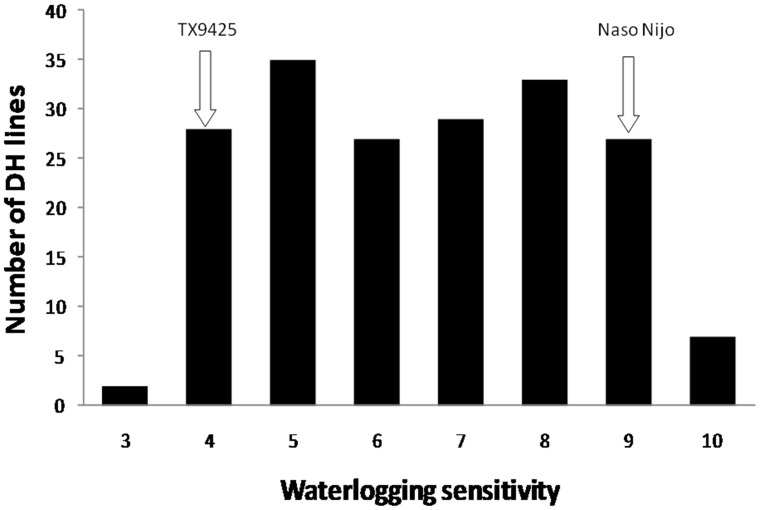
Frequency distribution for waterlogging tolerance at the end of waterlogging treatment of the DH lines derived from the cross of TX9425/Naso Nijo.

### Statistical Analysis

The average values from each experiment were used for the identification of QTLs associated with salt tolerance. Using the software package MapQTL5.0 [34], QTLs were first analysed by interval mapping (IM), followed by composite interval mapping (CIM). The closest marker at each putative QTL identified using interval mapping was selected as a cofactor and the selected markers were used as genetic background controls in the approximate multiple QTL model (MQM) of MapQTL5.0. Logarithm of the odds (LOD) threshold values applied to declare the presence of a QTL were estimated by performing the genome wide permutation tests implemented in MapQTL version 5.0 using at least 1000 permutations of the original data set for each trait, resulting in a 95% LOD threshold around 3.1. Two LOD support intervals around each QTL were established, by taking the two positions, left and right of the peak, that had LOD values of two less than the maximum [34], after performing restricted MQM mapping which does not use markers close to the QTL. The percentage of variance explained by each QTL (R^2^) was obtained using restricted MQM mapping implemented with MapQTL5.0. Graphical representation of linkage groups and QTL was carried out using MapChart 2.2 [35].

**Table 2:** QTL for salinity and waterlogging tolerance identified in the DH population of TX9425 × Naso Nijo.

QTL	Linkage group	Nearest marker	Position (cM)	LOD	R^2^ (%)
*QSl.TxNn.2H*	2H	bPb-6792	14.7	24.37	45.0
*QWl.TxNn.2H*	2H	bPb-5363	17.3	9.93	16.0
*QWl.TxNn.4H*	4H	GBM1501	0.0	5.48	8.4
*QWl.TxNn.5H*	5H	bPb-5845	144.7	4.87	7.4
*QWl.TxNn.7H*	7H	Ebmac0755	171.3	7.68	12.0

**Figure 4:**
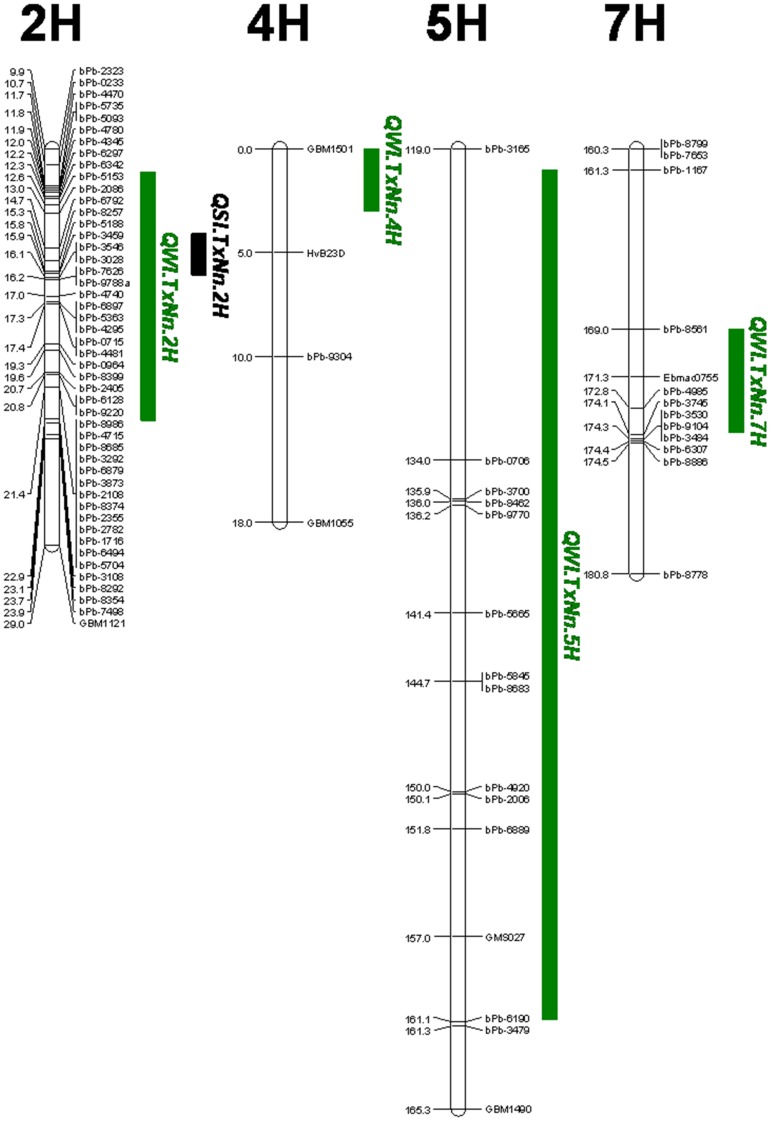
QTLs associated with salinity tolerance (in black) and waterlogging tolerance (in green). For clarity, only part of the chromosome regions which cover 2-LOD interval of all the QTLs are shown.

## Results

### Salinity and Waterlogging Tolerance of Parental and DH Lines

Two weeks after salt stress was applied, the lower leaves of susceptible varieties started to wilt which is a sign of salt damage. Wilting and leaf chlorosis increased with extended treatment time. The leaves of Naso Nijo finally died while TX9425 remained relatively healthy as the stress continued to the booting stage which was seven weeks after salt treatment. In the hydroponic experiment, Naso Nijo also showed much severe leaf chlorosis. DH lines showed significant differences in the damage caused by salinity stress (Figure 1). Figure 2 shows the frequency distribution of salinity tolerance for the 188 lines at the booting stage from the potting mixture trial. Statistical analysis identified the differences between the DH lines to be highly significant (Table 1). The two parents and the DH lines also showed significant differences in hydroponic experiment.

**Figure 5:**
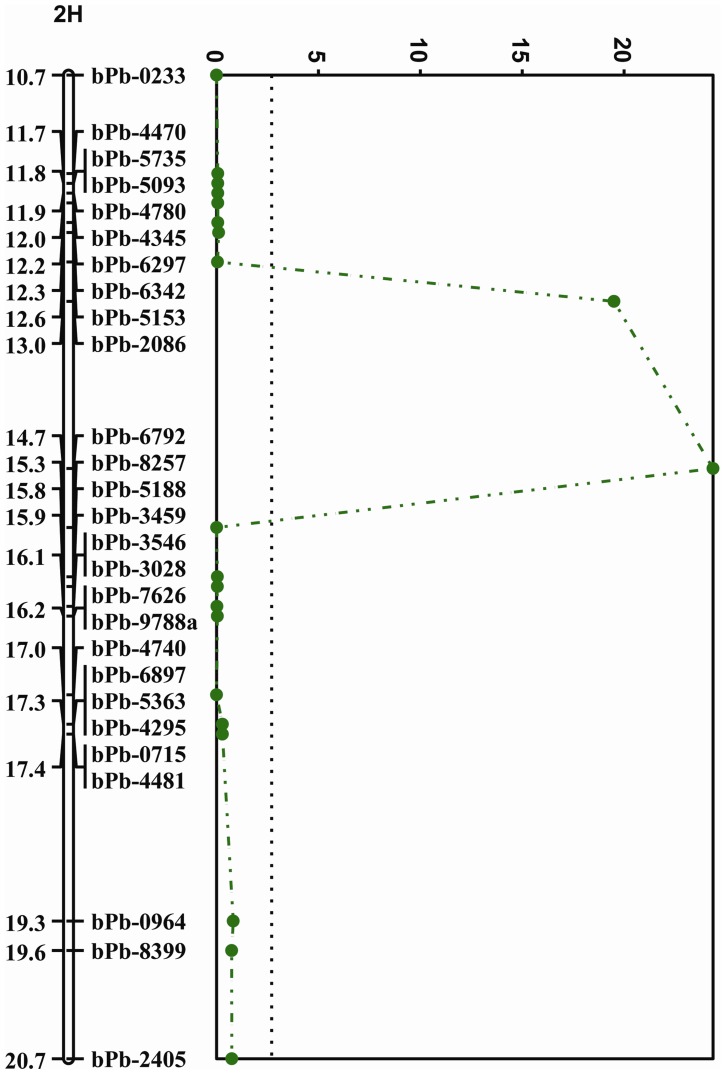
The QTL associated with salinity tolerance (LOD values). Only part of 2H was presented. The dotted line at LOD 3.1 is a line of significance.

TX9425 showed moderate but significantly better waterlogging tolerance than Naso Nijo (Figure 3). By the end of waterlogging treatment, TX9425 survived while most of Naso Nijo plants died. DH lines from the cross between TX9425 and Naso Nijo also showed significant differences in waterlogging tolerance (Table 1). Figure 3 shows the frequency distribution of waterlogging tolerance for the 188 lines at the end of waterlogging treatment with a near even distribution between moderately tolerant and very sensitive.

**Table 3:** QTL identified for different traits under salinity and control conditions in the DH population of TX9425 × Naso Nijo from hydroponic screening.

QTL	Linkage group	Nearest marker	Position (cM)	LOD	R^2^ (%)
PHC*	3H	65.6	Bmac0209	4.91	9.7
	7H	107.8	bPb-1050	4.30	8.5
	5H	72.2	bPb-9306	3.49	6.8
PHS	2H	22.9	bPb-3108	4.86	9.6
	3H	65.6	Bmac0209	4.86	9.6
	5H	75.8	Bmag0751	3.03	5.9
PHS/C	2H	22.9	bPb-3108	3.26	7.7
NoLC	–	–	–	–	–
NoLS	–	–	–	–	–
NoLS/C	–	–	–	–	–
NoTC	–	–	–	–	–
NoTS	2H	23.1	bPb-8292	3.12	7.3
NoTS/C	–	–	–	–	–
GSFWC	4H	21.4	bPb-3894	3.90	9.1
GSFWS	2H	22.9	bPb-3108	4.39	10.5
GSFWS/C	–	–	–	–	–
GSDWC	4H	21.4	bPb-3894	4.20	9.2
	3H	65.6	Bmac0209	3.07	6.6
GSDWS	2H	23.1	bPb-8292	9.17	18.1
	1H	66.5	Bmag0504	3.69	6.8
	7H	104.3	Bmc0187	3.08	5.6
GSDWS/C	2H	16.1	bPb-3546	4.17	9.7
YSFW	–	–	–	–	–
YSDW	–	–	–	–	–
NoYL	2H	20.7	bPb-2405	7.36	15.1
	3H	65.6	Bmac0209	3.58	7.0
RL	–	–	–	–	–
RFW	2H	14.7	bPb-6792	10.99	23.6
RDW	2H	11.7	bPb-4470	12.42	23.2
	3H	137.8	bPb-8506	4.02	6.8
	7H	174.5	bPb-8886	3.47	5.8
RFW/P	2H	22.9	bPb-3108	4.58	10.6
RDW/P	2H	16.1	bPb-3546	4.11	9.6
ALLDY	–	–	–	–	–

* See M&M for abbreviations.

### QTLs Associated with Waterlogging and Salinity Tolerance

Four QTLs were found to be associated with waterlogging tolerance. These are designated as *QWl.TxNn.2H, QWl.TxNn.4H*, *QWl.TxNn.5H* and *QWl.TxNn.7H*, explained a total of nearly 44% of the phenotypic variation (Table 2, Figure 4). Among these four QTLs, TX9425 contributed the tolerance to three of them (*QWl.TxNn.2H, QWl.TxNn.4H* and *QWl.TxNn.7H*) while Naso Nijo contributed the tolerance to *QWl.TxNn.5H*.

In contrast to waterlogging tolerance, only one significant QTL, *QSl.TxNn.2H*, was identified for salinity tolerance in the potting mixture trial. This QTL was located on chromosome 2H at a position of 14.7 cM. The single QTL explained 45% of the phenotypic variation. The closet marker is bPb-6792 (Table 2, Figures 4 and 5) with TX9425 contributing the tolerance.

In the hydroponic experiment, different traits were used to indicate the tolerance. Table 3 shows that QTLs were identified for most of the traits measured except for the number of leaves at harvest, yellow leaf fresh and dry weight, and root length under salinity treatment. Significant QTLs were identified for relative plant height, relative green leaf dry weight, number of yellow leaves, root fresh and dry weight, and plant healthiness score, explaining 7 to 24% of the phenotypic variation. The major QTL for these traits were all located on a similar position of *QSl.TxNn.2H*. Except for the major QTL, one minor QTL on 3H for the number of yellow leaves and two minor QTLs on 3H and 7H for root dry weight were also identified. One minor QTL was found for the number of tillers and green leaf fresh weight under salinity treatment, which also located on the similar position of *QSl.TxNn.2H*, but no significant QTL was identified for the relative values of both traits. No QTL was identified for the number of leaves and root length at the stage of harvest.

## Discussion

### Screening for Salinity and Waterlogging Tolerance

Many different methods have been used to screen plants for salt tolerance. Small scale screening systems, glasshouse trials and hydroponic methods have been developed to minimize the genetic by environmental interactions commonly encountered in field trials [12], [14], [16], [36], [37], [38], [39], [40], [41]. Shoot fresh and dry weights, as well as percent mortality, are common criteria for assessing relative salt tolerance. Two different screening systems were used in the current study, i.e. potting mixture [23] using plant survival and leaf chlorosis as criteria and the hydroponic system using various traits as salt tolerance indices. The facility with potting mixture proved to be very effective [23] and could clearly separate tolerant lines from sensitive ones in the DH population used in this study (Figure 1). In the hydroponic experiment, both parents and DH lines showed significant difference for most of salt tolerance related traits, but the differences were not as obvious as those in potting mixture experiment.

The screening system for waterlogging tolerance has successfully identified QTLs contributing waterlogging tolerance in several DH populations [26]. TX9425 showed much better tolerance to waterlogging than Naso Nijo but was relatively less tolerant than Yerong [26] and YYXT [42]. Not surprisingly, the average tolerance of the DH population for the cross of TX9425/Naso Nijo was not as good as that for the Yerong/Franklin and YYXT/Franklin populations.

### Molecular Markers for Waterlogging and Salinity Tolerance in Barley

Very slow progress has been made to introduce waterlogging and salinity tolerance into commercial varieties. One of the reasons is the lack of a reliable screening method to accurately identify the tolerance genes in the large breeding populations. The screening systems used in this study for waterlogging tolerance has been proved to be very effective in accurately allocating QTLs for the tolerance [26]. Four QTLs were identified in this population and TX9425 contributed tolerance to most of the QTLs. These QTLs were located on different positions from those found from other population in our earlier reports [19], [26] by allocating markers to consensus maps [32], [33], [43]. The result from the present study provides new genetic resources and QTL for further improvement of waterlogging tolerance by integrating of the QTLs identified in the previous studies [19], [26].

A large number of QTLs for salt tolerance have been identified in barley using different traits as tolerance indicators. Those included germination, chlorophyll content, chlorophyll florescence (F, Fv, Fm/Fv), tissue proline and carbohydrate content, relative water content, coleoptile and radicle length, wet and dry weights of tissues and shoot sodium content [11], [12], [16], [24]. In our potting mixture experiment, one single QTL was identified on 2H. The position with the nearest marker of bPb-6792 which is located at 17.7 cM of the consensus map [32] is different from previously reported QTLs on 2H with the nearest marker of bPb-5629 which is located at 56.2 cM of the consensus map [23], [32]. In the current hydroponic experiment, various traits have been used to indicating the tolerance to salinity. The QTL on 2H, which was found for the tolerance in the potting mixture experiment, was identified for most of the traits. From the percentage of phenotypic variation explained by the QTL, the number of yellow leaves, root fresh and dry weight, and the score for overall healthiness under salt treatment were shown to be relatively better criteria for evaluating salt tolerance. Using different consensus maps [32], [33], [43] the QTL identified for different traits can be compared with the results from previous reports.

The evaluation of salinity tolerance with potting mixture was again shown to be very effective in identifying QTL for salinity tolerance. In general, various stress tolerances are always controlled by several QTLs. The large numbers of QTL with relative small effect presents a challenge to effectively combine the QTLs through marker-assisted selection in the breeding programs. In this population, only one single significant QTL was identified and it explained 45% of the phenotypic variation. This makes it possible to accelerate transferring the tolerance gene into commercial varieties through MAS and further fine map the QTL and leads to possible cloning of the gene.

A consensus map for the salinity tolerance QTL region on 2H was constructed by JoinMap. Among all the markers, eight have DNA sequences. The molecular marker sequences were used to BLAST the rice and *Brachypodium* genome sequences (Table S1). Table S1 listed candidate genes in *Brachypodium* and rice genomes syntenic to salinity tolerance QTL on barley chromosome 2H. In total, the salinity tolerance QTL region contains 113 *Brachypodium* and 110 rice annotated genes, and the genes are ordered based on the Barley Genome Zipper [44]. The top border of the QTL could be clearly defined to syntenic regions of the rice chromosome 4 and *Brachypodium* chromosome 5. The bottom border may locate in a translocation region of rice chromosomes 4 and 7 (Table S1). Several transporter genes, e.g.Bradi5g02750, Bradi5g02520 and Bradi5g25010, or vacuolar-sorting receptor gene Bradig17670 may be the candidate genes for the salinity tolerance in this QTL. However, some key molecular markers including gene-specific SNP markers in the QTL region could not identify the syntenic genes in either rice or *Brachypodium* syntenic regions. It is likely that the QTL region identified in this cross for salinity tolerance may have totally different genes comparing to rice or *Brachypodium* genes. More research is required to fine-mapping the gene and validating of the gene functions.

The QTL on 2H for both waterlogging and salinity tolerance were located in a similar position, indicating some common physiological mechanisms for the tolerance. These physiological mechanisms include plant Na^+^ and Cl^−^ concentrations [45], [46], K^+^ uptake under salinity [14], [47] and waterlogging [18] conditions. Further study will be on allocating QTLs for different physiological traits and to compare them with that for salinity and waterlogging tolerance.

In conclusion, four QTLs were identified for waterlogging tolerance with most of them being different from those previously reported. Most importantly, only one single QTL was responsible for salinity tolerance from TX9425, a Chinese landrace variety. The markers linked to this QTL should be very effectively used in breeding program. This also paves a way for further fine mapping and cloning of the gene.

## Supporting Information

Table S1 **Candidate genes in** ***Brachypodium*** **and rice genomes syntenic to salinity tolerance QTL on barley chromosome 2H.**
(DOCX)
