# Supplementary material for: A Single Locus Is Responsible for Salinity Tolerance in a Chinese Landrace Barley (Hordeum vulgare L.)
Source: PLoS One. 2012 Aug 20;7(8):e43079. doi: 10.1371/journal.pone.0043079 (PMC3423432; doi:10.1371/journal.pone.0043079)
Supplement: Table S1 — Candidate genes in Brachypodium and rice genomes syntenic to salinity tolerance QTL on barley chromosome 2H. (DOCX) [file pone.0043079.s001.docx]

**Table S1** Candidate genes in *Brachypodium* and rice genomes syntenic to salinity tolerance QTL on barley chromosome 2H.

| Brachypodium genes | Brachypodium chromosome | Rice genes | Rice chromosome | Annotation in Brachypodium or rice |
| --- | --- | --- | --- | --- |
| Bradi5g01070.1 | 5 | - |  | "expressed protein" |
| Bradi5g01080.1 | 5 | - |  | "expressed protein" |
| Bradi5g01110.1 | 5 | - |  | "expressed protein" |
| - |  | Os04g0142700 | 4 |  |
| - |  | Os04g0141200 | 4 |  |
| - |  | Os04g0140400 | 4 |  |
| Bradi5g01135.1 | 5 | Os04g0137100 | 4 | "pectate lyase precursor, putative, expressed" |
| - |  | Os04g0136900 | 4 |  |
| Bradi5g01160.1 | 5 | - |  | "LIM domain-containing protein, putative, expressed" |
| Bradi5g01167.1 | 5 | - |  | "disease resistance protein RPM1, putative, expressed" |
| Bradi5g01210.1 | 5 | Os04g0132500 | 4 | "receptor-like protein kinase 2 precursor, putative, expressed" |
| Bradi5g01220.2 | 5 | Os04g0132300 | 4 | expressed protein |
| Bradi5g01230.1 | 5 | Os04g0131900 | 4 | "sterol 3-beta-glucosyltransferase, putative, expressed" |
| Bradi5g01240.1 | 5 | - |  | "transferase family protein, putative, expressed" |
| Bradi5g01270.1 | 5 | Os04g0129600 | 4 | UDP-glucosyltransferase 74F2 |
| Bradi5g01280.1 | 5 | Os04g0129500 | 4 | "protein transport protein Sec24-like, putative, expressed" |
| Bradi5g01310.2 | 5 | - |  | expressed protein |
| Bradi5g01317.1 | 5 | Os04g0129300 | 4 | "ECT protein, putative, expressed" |
| Bradi5g01327.1 | 5 | - |  | "flavin monooxygenase, putative, expressed" |
| Bradi5g01340.1 | 5 | Os04g0129200 | 4 | "3-5 exonuclease domain-containing protein, putative, expressed" |
| - |  | Os04g0128900 | 4 |  |
| - |  | Os04g0125700 | 4 |  |
| Bradi5g01350.1 | 5 | - |  | "VHS and GAT domain containing protein, expressed" |
| Bradi5g01400.1 | 5 | - |  | "cytochrome P450, putative, expressed" |
| Bradi5g01410.1 | 5 | Os04g0119500 | 4 | "membrane related protein CP5, putative, expressed" |
| Bradi5g01420.1 | 5 | Os04g0119400 | 4 | "dehydrogenase E1 component domain containing protein, expressed" |
| Bradi5g01430.1 | 5 | Os04g0118900 | 4 | "RNA recognition motif containing protein, putative, expressed" |
| - |  | Os04g0118500 | 4 | PIF1 helicase |

**Table S1** Candidate genes in *Brachypodium* and rice genomes syntenic to salinity tolerance QTL on barley chromosome 2H (continued).

| Brachypodium genes | Brachypodium chromosome | Rice genes | Rice chromosome | Annotation in Brachypodium or rice |
| --- | --- | --- | --- | --- |
| Bradi5g01470.1 | 5 | - |  | "NB-ARC domain containing protein, expressed" |
| Bradi5g01480.1 | 5 | - |  | "NB-ARC domain containing protein, expressed" |
| Bradi5g01567.1 | 5 | - |  | "protein kinase domain containing protein, expressed" |
| Bradi5g00750.1 | 5 | - |  | "transferase family protein, putative, expressed" |
| - |  | Os04g0175500 | 4 |  |
| - |  | Os04g0176400 | 4 |  |
| Bradi5g00770.1 | 5 | Os04g0177300 | 4 | "SWI/SNF-related matrix-associated actin-dependent regulator of chromatin subfamily A, putative, expressed" |
| - |  | Os04g0177400 | 4 |  |
| Bradi5g00777.1 | 5 | Os04g0177600 | 4 | "actin, putative, expressed" |
| - |  | Os04g0178300 | 4 |  |
| - |  | Os04g0178400 | 4 |  |
| - |  | Os04g0179700 | 4 |  |
| Bradi5g00800.1 | 5 | - |  | "1-aminocyclopropane-1-carboxylate oxidase homolog 2, putative, expressed" |
| Bradi5g00810.1 | 5 | - |  | "12-oxophytodienoate reductase, putative, expressed" |
| - |  | Os04g0180400 | 4 |  |
| Bradi5g00830.1 | 5 | Os04g0182200 | 4 | "1-aminocyclopropane-1-carboxylate oxidase homolog 2, putative, expressed" |
| Bradi5g00840.1 | 5 | - |  | "OsSub40 - Putative Subtilisin homologue, expressed" |
| Bradi5g00847.1 | 5 | - |  | "glycine-rich protein, putative, expressed" |
| Bradi5g00860.1 | 5 | Os04g0182900 | 4 | "amidase family protein, putative, expressed" |
| - |  | Os04g0183100 | 4 |  |
| - |  | Os04g0183300 | 4 |  |
| - |  | Os04g0183500 | 4 |  |
| Bradi5g00890.1 | 5 | - |  | "amidase, putative, expressed" |
| Bradi5g01720.1 | 5 | - |  | "peptidyl-prolyl cis-trans isomerase, putative, expressed" |
| - |  | Os04g0118100 | 4 | HXXXD-type acyl-transferase family protein |
| Bradi5g01730.1 | 5 | Os04g0117600 | 4 | "tRNA-dihydrouridine synthase 3-like, putative, expressed" |

**Table S1** Candidate genes in *Brachypodium* and rice genomes syntenic to salinity tolerance QTL on barley chromosome 2H (continued).

| Brachypodium genes | Brachypodium chromosome | Rice genes | Rice chromosome | Annotation in Brachypodium or rice |
| --- | --- | --- | --- | --- |
| Bradi5g01737.1 | 5 | - |  | "peptide transporter PTR2, putative, expressed" |
| Bradi5g01760.1 | 5 | Os04g0117200 | 4 | expressed protein |
| Bradi5g01763.1 | 5 | Os04g0117100 | 4 | expressed protein |
| Bradi5g01766.1 | 5 | - |  | "terpene synthase, putative, expressed" |
| Bradi5g01770.1 | 5 | - |  | "transferase family protein, putative, expressed" |
| Bradi5g01823.1 | 5 | - |  | "terpene synthase, putative, expressed" |
| Bradi5g01836.1 | 5 | - |  | "hexokinase, putative, expressed" |
| Bradi5g01850.1 | 5 | Os04g0116900 | 4 | expressed protein |
| - |  | Os04g0115500 | 4 |  |
| Bradi5g01880.1 | 5 | Os04g0115400 | 4 | "G-patch domain containing protein, expressed" |
| Bradi5g01890.1 | 5 | Os04g0112300 | 4 | "tRNA methyltransferase, putative, expressed" |
| Bradi5g01942.1 |  | - |  | "transposon protein, putative, unclassified, expressed" |
| Bradi5g01950.1 |  | - |  | "bifunctional 3-phosphoadenosine 5-phosphosulfate synthetase, putative, expressed" |
| - |  | Os04g0111200 | 4 |  |
| Bradi5g01960.1 | 5 | Os04g0110600 | 4 | "zinc finger family protein, putative, expressed" |
| - |  | Os04g0110500 | 4 |  |
| - |  | Os04g0109100 | 4 |  |
| Bradi5g01970.1 | 5 | - |  | "receptor protein kinase, putative, expressed" |
| Bradi5g02030.1 | 5 | - |  | "white-brown complex homolog protein 11, putative, expressed" |
| Bradi5g02037.1 | 5 | - |  | "heat shock protein, putative, expressed" |
| Bradi5g02060.3 | 5 | - |  | expressed protein |
| Bradi5g02090.1 | 5 | Os04g0107500 | 4 | "erythronate-4-phosphate dehydrogenase domain containing protein, expressed" |
| Bradi5g02100.1 | 5 | Os04g0107200 | 4 | "erythronate-4-phosphate dehydrogenase domain containing protein, expressed" |
| Bradi5g02120.1 | 5 | - |  | "RNA recognition motif containing protein, putative, expressed" |
| Bradi5g02130.1 | 5 | - |  | "erythronate-4-phosphate dehydrogenase domain containing protein, expressed" |

**Table S1** Candidate genes in *Brachypodium* and rice genomes syntenic to salinity tolerance QTL on barley chromosome 2H (continued).

| Brachypodium genes | Brachypodium chromosome | Rice genes | Rice chromosome | Annotation in Brachypodium or rice |
| --- | --- | --- | --- | --- |
| Bradi5g02150.1 | 5 | - |  | "erythronate-4-phosphate dehydrogenase domain containing protein, expressed" |
| - |  | Os04g0106400 | 4 | "Amidase family protein" |
| Bradi5g02160.1 | 5 | Os04g0106300 | 4 | "arginase, putative, expressed" |
| Bradi5g02170.1 | 5 | - |  | "acetyl-CoA acetyltransferase, cytosolic, putative, expressed" |
| Bradi5g02190.1 | 5 | Os04g0105400 | 4 | expressed protein |
| Bradi5g02200.1 | 5 | - |  | "adenylate kinase, putative, expressed" |
| Bradi1g17610.1 | 1 | - |  | "HAD superfamily phosphatase, putative, expressed" |
| - |  | Os07g0681200 | 7 |  |
| Bradi1g17620.1 | 1 | Os07g0681100 | 7 | "inactive receptor kinase At2g26730 precursor, putative, expressed" |
| - |  | Os07g0680900 | 7 |  |
| - |  | Os07g0681000 | 7 |  |
| Bradi1g17630.1 | 1 | - |  | "AGC_AGC_other_RS6K_like.2 - ACG kinases include homologs to PKA, PKG and PKC, expressed" |
| Bradi1g17650.1 | 1 | Os07g0680500 | 7 | "SNF2 family N-terminal domain containing protein, expressed" |
| Bradi1g17660.1 | 1 | Os07g0680400 | 7 | "WRKY47, expressed" |
| Bradi1g17665.1 | 1 | Os07g0680300 | 7 | "ubiquinol-cytochrome c reductase complex 6.7 kDa protein, putative, expressed" |
| Bradi1g17670.1 | 1 | Os07g0680000 | 7 | "vacuolar-sorting receptor precursor, putative, expressed" |
| Bradi1g17680.1 | 1 | Os07g0679700 | 7 | "B3 DNA binding domain containing protein, putative, expressed" |
| Bradi1g17700.1 | 1 | Os07g0679500 | 7 | "bZIP transcription factor domain containing protein, expressed" |
| Bradi1g17710.1 | 1 | - |  | "HEAT repeat family protein, putative, expressed" |
| Bradi1g17720.1 | 1 | - |  | "nucleotidyltransferase, putative, expressed" |
| Bradi1g17730.1 | 1 | Os07g0679300 | 7 | "alpha-galactosidase precursor, putative, expressed" |
| Bradi1g17750.1 | 1 | - |  | "OsFBX287 - F-box domain containing protein, expressed" |
| Bradi1g17755.1 | 1 | - |  | "OsFBX287 - F-box domain containing protein, expressed" |

**Table S1** Candidate genes in *Brachypodium* and rice genomes syntenic to salinity tolerance QTL on barley chromosome 2H (continued).

| Brachypodium genes | Brachypodium chromosome | Rice genes | Rice chromosome | Annotation in Brachypodium or rice |
| --- | --- | --- | --- | --- |
| Bradi5g03110.1 | 5 | - |  | "disease resistance protein RPM1, putative, expressed" |
| Bradi5g03077.1 | 5 | - |  | "OsFBLD6 - F-box, LRR and FBD domain containing protein, expressed" |
| Bradi5g03060.1 | 5 | - |  | "OsWAK104 - OsWAK receptor-like protein kinase, expressed" |
| Bradi5g02990.1 | 5 | - |  | "peptidyl-prolyl cis-trans isomerase, FKBP-type, putative, expressed" |
| Bradi5g02980.1 | 5 | - |  | "receptor-like protein kinase, putative, expressed" |
| Bradi5g02967.1 | 5 | - |  | "reticuline oxidase-like protein precursor, putative, expressed" |
| Bradi5g02950.1 | 5 | - |  | "reticuline oxidase-like protein precursor, putative, expressed" |
| Bradi5g02940.2 | 5 | - |  | "transcriptional regulator Sir2 family protein, putative, expressed" |
| Bradi5g02920.1 | 5 | - |  | "transmembrane amino acid transporter protein, putative, expressed" |
| - |  | Os04g0203600 | 4 | "UDP-glycosyltransferase 73B4" |
| - |  | Os04g0203500 | 4 |  |
| - |  | Os04g0202800 | 4 | "Protein-tyrosine phosphatase-like, PTPLA" |
| - |  | Os04g0202500 | 4 |  |
| - |  | Os04g0202300 | 4 | "Uncharacterised protein family (UPF0172)" |
| - |  | Os04g0201900 | 4 |  |
| - |  | Os04g0201800 | 4 |  |
| - |  | Os04g0201500 | 4 |  |
| Bradi5g02912.1 | 5 | Os04g0201200 | 4 | "pumilio-family RNA binding repeat containing protein, expressed" |
| - |  | Os04g0201000 | 4 |  |
| Bradi5g02906.1 | 5 | - |  | "OsFBX118 - F-box domain containing protein, expressed" |
| Bradi5g02890.3 | 5 | - |  | "T-complex protein, putative, expressed" |
| - |  | Os04g0197200 | 4 |  |
| Bradi5g02870.1 | 5 | Os04g0194500 | 4 | "white-brown complex homolog protein, putative, expressed" |
| - |  | Os04g0193300 | 4 |  |
| Bradi5g02860.1 | 5 | - |  | "disease resistance RPP13-like protein 1, putative, expressed" |
| - |  | Os04g0191800 | 4 |  |

**Table S1** Candidate genes in *Brachypodium* and rice genomes syntenic to salinity tolerance QTL on barley chromosome 2H (continued).

| Brachypodium genes | Brachypodium chromosome | Rice genes | Rice chromosome | Annotation in Brachypodium or rice |
| --- | --- | --- | --- | --- |
| - |  | Os04g0191000 | 4 |  |
| Bradi5g02840.1 | 5 | Os04g0189400 | 4 | "gamma-thionin family domain containing protein, expressed" |
| Bradi5g02830.2 | 5 | - |  |  |
| Bradi5g02820.1 | 5 | - |  |  |
| Bradi5g02800.1 | 5 | Os04g0188400 | 4 | "expressed protein" |
| Bradi5g02780.1 | 5 | Os04g0206700 | 4 | "UDP-glucoronosyl/UDP-glucosyl transferase, putative, expressed" |
| - |  | Os04g0186400 | 4 |  |
| Bradi5g02750.1 | 5 | Os04g0186800 | 4 | "inorganic phosphate transporter, putative, expressed" |
| Bradi5g02520.1 | 5 | - |  | "transporter family protein, putative, expressed" |
| Bradi5g02510.1 | 5 | - |  | "nuclear transport factor, putative, expressed" |
| - |  | Os07g0456700 | 7 |  |
| - |  | Os07g0457300 | 7 |  |
| - |  | Os07g0458900 | 7 |  |
| - |  | Os07g0459400 | 7 |  |
| - |  | Os07g0460801 | 7 |  |
| - |  | Os07g0461500 | 7 |  |
| - |  | Os07g0461700 | 7 | "rhomboid protein-related" |
| - |  | Os07g0462200 | 7 | "SNARE associated Golgi protein family" |
| - |  | Os07g0463600 | 7 | "Lactoylglutathione lyase / glyoxalase I family protein" |
| - |  | Os07g0467500 | 7 | "Glutathione S-transferase, C-terminal-like;Translation elongation factor EF1B/ribosomal protein S6" |
| - |  | Os07g0467600 | 7 | "RNA-binding (RRM/RBD/RNP motifs) family protein" |
| - |  | Os07g0467900 | 7 | "disproportionating enzyme 2" |
| - |  | Os07g0472500 | 7 | "alpha/beta-Hydrolases superfamily protein" |
| - |  | Os07g0474300 | 7 |  |
| Bradi5g02500.1 | 5 | Os07g0474400 | 7 | "mycolic acid methyl transferase, putative, expressed" |
| - |  | Os07g0474600 | 7 | "SKP1 interacting partner 6" |
| - |  | Os07g0475700 | 7 |  |
| - |  | Os07g0476500 | 7 | "SOS3-interacting protein 4" |
| - |  | Os07g0479200 | 7 |  |
| - |  | Os07g0480900 | 7 | "apyrase 2" |
| - |  | Os07g0484200 | 7 |  |

**Table S1** Candidate genes in *Brachypodium* and rice genomes syntenic to salinity tolerance QTL on barley chromosome 2H (continued).

| Brachypodium genes | Brachypodium chromosome | Rice genes | Rice chromosome | Annotation in Brachypodium or rice |
| --- | --- | --- | --- | --- |
| - |  | Os07g0484300 | 7 |  |
| Bradi5g02490.1 | 5 | - |  | "Cyclopropane-fatty-acyl-phospholipid synthase, putative, expressed" |
| Bradi5g02480.1 | 5 | - |  | "transposon protein, putative, CACTA, En/Spm sub-class, expressed" |
| - |  | Os04g0100300 | 4 |  |
| Bradi5g02470.2 | 5 | Os04g0101300 | 4 | "chromatin modification-related protein EAF3, putative, expressed" |
| Bradi5g02460.1 | 5 | Os04g0101400 | 4 | "cytochrome P450 93A2, putative, expressed" |
| Bradi5g02447.1 | 5 | Os04g0101700 | 4 | "ELMO/CED-12 family protein, putative, expressed" |
| Bradi5g02430.1 | 5 | - |  | "Leucine Rich Repeat family protein, expressed" |
| Bradi5g02400.3 | 5 | Os04g0102500 | 4 | "phosphoglycerate mutase, putative, expressed" |
| Bradi5g02390.1 | 5 | Os04g0102600 | 4 | "serine-type peptidase, putative, expressed" |
| Bradi5g02380.1 | 5 | Os04g0102700 | 4 | "amidase family protein, putative, expressed" |
| Bradi5g02240.1 | 5 | - |  | "Leucine Rich Repeat family protein, expressed" |
| Bradi5g02250.1 | 5 | - |  | "serpin domain containing protein, putative, expressed" |
| Bradi5g02270.1 | 5 | Os04g0105300 | 4 | "DUF1295 domain containing protein, putative, expressed" |
